# Supplementary material for: Bayesian refinement of protein structures and ensembles against SAXS data using molecular dynamics
Source: PLoS Comput Biol. 2017 Oct 18;13(10):e1005800. doi: 10.1371/journal.pcbi.1005800 (PMC5662244; doi:10.1371/journal.pcbi.1005800)
Supplement: S1 Archive — (BZ2) [file pcbi.1005800.s011.tar.bz2 › gromacs_4.62_bayesian_saxs_refi/share/html/online/g_bar.html]

g\_bar


| g\_bar Main Table of Contents |

**VERSION 4.6  
Sat 19 Jan 2013**

---

### Description

g\_bar calculates free energy difference estimates through
Bennett's acceptance ratio method (BAR). It also automatically
adds series of individual free energies obtained with BAR into
a combined free energy estimate.

Every individual BAR free energy difference relies on two
simulations at different states: say state A and state B, as
controlled by a parameter, λ (see the .mdp parameter
init\_lambda). The BAR method calculates a ratio of weighted
average of the Hamiltonian difference of state B given state A and
vice versa.
The energy differences to the other state must be calculated
explicitly during the simulation. This can be done with
the .mdp option foreign\_lambda.

Input option -f expects multiple dhdl.xvg files.
Two types of input files are supported:  
\* Files with more than one y-value.
The files should have columns
with dH/dλ and Δλ.
The λ values are inferred
from the legends: λ of the simulation from the legend of
dH/dλ and the foreign λ values from the
legends of Delta H
  
\* Files with only one y-value. Using the
-extp option for these files, it is assumed
that the y-value is dH/dλ and that the
Hamiltonian depends linearly on λ.
The λ value of the simulation is inferred from the
subtitle (if present), otherwise from a number in the subdirectory
in the file name.

The λ of the simulation is parsed from
dhdl.xvg file's legend containing the string 'dH', the
foreign λ values from the legend containing the
capitalized letters 'D' and 'H'. The temperature is parsed from
the legend line containing 'T ='.

The input option -g expects multiple .edr files.
These can contain either lists of energy differences (see the
.mdp option separate\_dhdl\_file), or a series of
histograms (see the .mdp options dh\_hist\_size and
dh\_hist\_spacing).
The temperature and λ
values are automatically deduced from the ener.edr file.

In addition to the .mdp option foreign\_lambda,
the energy difference can also be extrapolated from the
dH/dλ values. This is done with the-extp
option, which assumes that the system's Hamiltonian depends linearly
on λ, which is not normally the case.

The free energy estimates are determined using BAR with bisection,
with the precision of the output set with -prec.
An error estimate taking into account time correlations
is made by splitting the data into blocks and determining
the free energy differences over those blocks and assuming
the blocks are independent.
The final error estimate is determined from the average variance
over 5 blocks. A range of block numbers for error estimation can
be provided with the options -nbmin and -nbmax.

g\_bar tries to aggregate samples with the same 'native' and
'foreign' λ values, but always assumes independent
samples. **Note** that when aggregating energy
differences/derivatives with different sampling intervals, this is
almost certainly not correct. Usually subsequent energies are
correlated and different time intervals mean different degrees
of correlation between samples.

The results are split in two parts: the last part contains the final
results in kJ/mol, together with the error estimate for each part
and the total. The first part contains detailed free energy
difference estimates and phase space overlap measures in units of
kT (together with their computed error estimate). The printed
values are:  
\* lam\_A: the λ values for point A.  
\* lam\_B: the λ values for point B.  
\* DG: the free energy estimate.  
\* s\_A: an estimate of the relative entropy of B in A.  
\* s\_A: an estimate of the relative entropy of A in B.  
\* stdev: an estimate expected per-sample standard deviation.

The relative entropy of both states in each other's ensemble can be
interpreted as a measure of phase space overlap:
the relative entropy s\_A of the work samples of lambda\_B in the
ensemble of lambda\_A (and vice versa for s\_B), is a
measure of the 'distance' between Boltzmann distributions of
the two states, that goes to zero for identical distributions. See
Wu & Kofke, J. Chem. Phys. 123 084109 (2005) for more information.

The estimate of the expected per-sample standard deviation, as given
in Bennett's original BAR paper: Bennett, J. Comp. Phys. 22, p 245 (1976).
Eq. 10 therein gives an estimate of the quality of sampling (not directly
of the actual statistical error, because it assumes independent samples).

To get a visual estimate of the phase space overlap, use the
-oh option to write series of histograms, together with the
-nbin option.

### Files

| option | filename | type | description |
| --- | --- | --- | --- |
| **-f** | dhdl.xvg | Input, Opt., Mult. | xvgr/xmgr file |
| **-g** | ener.edr | Input, Opt., Mult. | Energy file |
| **-o** | bar.xvg | Output, Opt. | xvgr/xmgr file |
| **-oi** | barint.xvg | Output, Opt. | xvgr/xmgr file |
| **-oh** | histogram.xvg | Output, Opt. | xvgr/xmgr file |

### Other options

| option | type | default | description |
| --- | --- | --- | --- |
| **-[no]h** | bool | no | Print help info and quit || **-[no]version** | bool | no | Print version info and quit || **-nice** | int | 0 | Set the nicelevel || **-[no]w** | bool | no | View output .xvg, .xpm, .eps and .pdb files || **-xvg** | enum | xmgrace | xvg plot formatting: xmgrace, xmgr or none || **-b** | real | 0 | Begin time for BAR || **-e** | real | -1 | End time for BAR || **-temp** | real | -1 | Temperature (K) || **-prec** | int | 2 | The number of digits after the decimal point || **-nbmin** | int | 5 | Minimum number of blocks for error estimation || **-nbmax** | int | 5 | Maximum number of blocks for error estimation || **-nbin** | int | 100 | Number of bins for histogram output || **-[no]extp** | bool | no | Whether to linearly extrapolate dH/dl values to use as energies |

---

http://www.gromacs.org  
gromacs@gromacs.org
